# Complete Chloroplast Genomes of *Anthurium huixtlense* and *Pothos scandens* (Pothoideae, Araceae): Unique Inverted Repeat Expansion and Contraction Affect Rate of Evolution

**DOI:** 10.1007/s00239-020-09958-w

**Published:** 2020-07-09

**Authors:** Abdullah, Claudia L. Henriquez, Furrukh Mehmood, Monica M. Carlsen, Madiha Islam, Mohammad Tahir Waheed, Peter Poczai, Thomas B. Croat, Ibrar Ahmed

**Affiliations:** 1 Department of Biochemistry, Faculty of Biological Sciences, Quaid-I-Azam University, Islamabad, 45320 Pakistan; 2 Department of Ecology and Evolutionary Biology, University of California, Los Angeles, Los Angeles, USA; 3 Missouri Botanical Garden, St. Louis, MO USA; 4 Department of Genetics, Hazara University, Mansehra, Pakistan; 5 Finnish Museum of Natural History, University of Helsinki, PO Box 7, 00014 Helsinki, Finland; 6 Alpha Genomics Private Limited, Islamabad, 45710 Pakistan

**Keywords:** Araceae, Pothoideae, *Pothos*, *Anthurium*, Inverted repeat contraction and expansion, Gene rearrangement, Gene evolution

## Abstract

The subfamily Pothoideae belongs to the ecologically important plant family Araceae. Here, we report the chloroplast genomes of two species of the subfamily Pothoideae: *Anthurium huixtlense* (size: 163,116 bp) and *Pothos scandens* (size: 164,719 bp). The chloroplast genome of *P. scandens* showed unique contraction and expansion of inverted repeats (IRs), thereby increasing the size of the large single-copy region (LSC: 102,956 bp) and decreasing the size of the small single-copy region (SSC: 6779 bp). This led to duplication of many single-copy genes due to transfer to IR regions from the small single-copy (SSC) region, whereas some duplicate genes became single copy due to transfer to large single-copy regions. The rate of evolution of protein-coding genes was affected by the contraction and expansion of IRs; we found higher mutation rates for genes that exist in single-copy regions as compared to those in IRs. We found a 2.3-fold increase of oligonucleotide repeats in *P. scandens* when compared with *A. huixtlense*, whereas amino acid frequency and codon usage revealed similarities. The ratio of transition to transversion mutations was 2.26 in *P. scandens* and 2.12 in *A. huixtlense*. Transversion mutations mostly translated in non-synonymous substitutions. The phylogenetic inference of the limited species showed the monophyly of the Araceae subfamilies. Our study provides insight into the molecular evolution of chloroplast genomes in the subfamily Pothoideae and family Araceae.

**Electronic supplementary material:**

The online version of this article (10.1007/s00239-020-09958-w) contains supplementary material, which is available to authorized users.

## Introduction

The plant family Araceae belongs to the order Alismatales. Araceae is a large and ancient monocot family that dates back to the Early Cretaceous period (Nauheimer et al. 2012). This family consists of 144 genera and 3645 species (Boyce and Croat 2018). It is the most diverse monocotyledon family in terms of morphology, encompassing the smallest known angiosperms (*Wolffia* Schkeid. species) as well as some of the plants having the largest vegetative and reproductive structures [*Amorphophallus titanum* (Becc) Becc. ex Arcang] (Gunawardena and Dengler 2006). Ecologically, species of Araceae are highly versatile, ranging from submerged, emergent or free-floating aquatics to epiphytic, climbing and terrestrial plants (Cabrera et al. 2008). Species of Araceae have been subdivided into eight subfamilies, distributed in tropical and temperate regions (Cabrera et al. 2008; Cusimano et al. 2011; Nauheimer et al. 2012). Pothoideae is the second largest subfamily, with approximately 1010 described species and approximately 2072 estimated species (Boyce and Croat 2018). The subfamily is divided into two tribes: Tribe Potheae which includes the genera *Pothos* L., *Pedicellarum* M.Hotta, and *Pothoidium* Schott, and the monogeneric Tribe Anthurieae comprised of species of *Anthurium* Schott (Mayo et al. 1997; Cabrera et al. 2008; Cusimano et al. 2011; Chartier et al. 2014). Members of Pothoideae are recognized by fine reticulate venation, complete lack of laticifers, usually aerial stems, apically geniculate petioles, bisexual perigoniate flowers, and a simple spathe not enclosing the spadix (Mayo et al. 1997). Pothoideae contains many climbing and hemiepiphytic species that live in humid tropical forests. The largest genera are *Pothos* and *Anthurium*, with approximately 57 and 950 described, and approximately 70 and 2000 estimated species, respectively (Boyce and Croat 2018). The other two genera, *Pothoidium* and *Pedicellarum*, are monospecific. *Pothos* is distributed in south and Southeast Asia, Australia, the Malagasy region, and the Malay Archipelago, while *Anthurium* is a strictly Neotropical genus that ranges from southern Mexico to southern Brazil, extending into the West Indies (Mayo et al. 1997; Carlsen and Croat 2013).

The chloroplast is a self-replicating organelle that plays a vital role in photosynthesis and in the synthesis of fatty acids and amino acids (Cooper 2000). In most plant lineages, the chloroplast contains its own circular double-stranded genome and has a primarily quadripartite structure in which a pair of long inverted repeat regions (IRa and IRb) separate the large single-copy (LSC) and small single-copy (SSC) regions (Palmer 1985). However, linear chloroplast genomes have also been reported (Oldenburg and Bendich 2016) in some species. Moreover, a quadripartite structure has not been observed in the chloroplast genomes of various species, such as Pinaceae (Wu et al. 2011), Cephalotaxaceae (Yi et al. 2013), and Taxodiaceae (Hirao et al. 2008). The size of the chloroplast genome of photosynthetic plants varies from 107 kb (*Cathaya argyrophylla* Chun & Kuang) to 218 kb (*Pelargonium x hortorum* L.H.Bailey) (Daniell et al. 2016). Chloroplast genomes are inherited from a single parent and show significant polymorphism (Daniell 2007; Daniell et al. 2016), which makes them well-suited for studies on phylogenetics and population genetics (Ahmed et al. 2013; Henriquez et al. 2014; Ahmed 2015).

Despite a relatively conserved structure, including gene organization, gene content, and intron content within genes (Iram et al. 2019; Mehmood et al. 2020; Shahzadi et al. 2020), chloroplast genomes have also undergone gene loss, intron loss, gene rearrangement, pseudogenization, gene duplication, and uneven expansion and contraction of IR regions. These events have led to a variable number of genes in the chloroplast genomes of angiosperms (Menezes et al. 2018; Abdullah et al. 2020b; Henriquez et al. 2020b). Moreover, the shifting of genes to single-copy regions from IR or vice versa due to IR contraction and expansion also affect the rate of DNA sequence evolution; the phenomenon is known as rate heterotachy (Lockhart et al. 2006). Previous studies of subfamilies Lemnoideae and Aroideae revealed unique and uneven contraction and expansion of IR regions, which led to a variable number of genes and gene rearrangements in the chloroplast genomes of several of their respective taxa (Wang and Messing 2011; Choi et al. 2017; Tian et al. 2018; Kim et al. 2019; Henriquez et al. 2020b). The aforementioned studies did not include species of the subfamily Pothoideae.

In this study, a comparison of the de novo assembled chloroplast genomes of *A. huixtlense* Matuda and *P. scandens* L. with chloroplast genomes of other Araceae species confirmed unique events of IR contraction and expansion in the chloroplast genome of *P. scandens*. The results revealed the transfer of IR genes to the LSC region at the junction of JLB (LSC/IRb) and the transfer of all SSC genes (except *rps*15 and *ycf*1) to the IR region at the junction of JSB (IRb/SSC). This transfer decreased the size of SSC region to 6779 bp and promotes heterotachy in Pothoideae by affecting the rate of evolution of these genes. To the best of our knowledge, the shortening of the SSC region to such an extent and the effects on genes evolution rate are reported here in Araceae for the first time. These results improve our understanding of the evolution of chloroplast genomes in Araceae.

## Materials and Methods

### DNA Extraction and Sequencing

We collected fresh healthy leaves of *P. scandens* and *A. huixtlense* from the Aroid Greenhouse at the Missouri Botanical Garden in St. Louis, Missouri. Total genomic DNA was extracted from these leaves using a Qiagen DNeasy Minikit (Qiagen, Germantown, Maryland, USA) following Henriquez et al. (2020a). Confirmation of the quality and quantity of DNA was performed using 1% gel electrophoresis and Nanodrop (ThermoScientific, Delaware, USA). Library preparation and sequencing were performed using TruSeq kits (Illumina, Inc., San Diego, California) in the Pires lab at the University of Missouri, Columbia following Henriquez et al. (2020a).

### De novo Assembly and Annotation

The quality of raw reads was analyzed by FastQC (Andrews 2017) and MultiQC (Ewels et al. 2016) for comparison. After quality confirmation, the Fast-Plast v. 1.2.2 pipeline (https://github.com/mrmckain/Fast-Plast) was initially used to assemble the raw reads following similar parameters previously employed for the assembly of chloroplast genomes of subfamilies Aroideae and Monsteroideae (Henriquez et al. 2020a,b). The resulting assembly from Fast-Plast was further confirmed by de novo assembly using Velvet v.1.2.10 following Abdullah et al. (2019a, 2020b) using Kmer values of 61, 71, and 81. Validation and coverage depth analyses of de novo assembled genomes were performed by mapping short reads to their respective assembled chloroplast genomes. The assembled chloroplast genomes were annotated using GeSeq (Tillich et al. 2017) and the circular diagrams of the annotated genomes were drawn using OrganellarGenomeDRAW (OGDRAW v.13.1) (Greiner et al. 2019). The five-column tab-delimited tables were generated for de novo assembled chloroplast genomes using GB2sequin (Lehwark and Greiner 2019) and were submitted to the National Center for Biotechnology Information (NCBI) under accession number MN046891 (*P. scandens*) and MN996266 (*A. huixtlense*). The raw reads were also submitted to the sequence read archive (SRA) of NCBI under BioProject number PRJNA547619.

### Characterization and Comparative Analyses of Chloroplast Genomes

Characterization of the chloroplast genomes of *P. scandens* and *A. huixtlense* and analyses of amino acid frequency and codon usage were performed in Geneious R8.1 (Kearse et al. 2012). Oligonucleotide repeats were determined using REPuter (Kurtz et al. 2001) by setting the parameter of minimum repeat size ≥ 30 and with minimum repeat similarity of 90%.

The chloroplast genome structure and gene content of *P. scandens* and *A. huixtlense* were compared with eight previously reported chloroplast genomes of Araceae, including *Anchomanes hookeri* (Kunth) Schott, *Anubias heterophylla* Engler, *Zantedeschia aethiopica* (L.) Spreng. (Henriquez et al. 2020b), *Epipremnum amplissimum* (Schott) Engl. (Henriquez et al. 2020a), *Spathiphyllum kochii* Engl. & K. Krause (Han et al. 2016), *Spirodela polyrrhiza* (L.) Schleid*.*, *Wolffiella lingulata* Hegelm. (Wang and Messing 2011), and *Symplocarpus renifolius* Schott ex Tzvelev (Choi et al. 2017). The gene content and rearrangement of the genome were compared by integrated Mauve alignment (Darling et al. 2004) in Geneious R8.1 based on collinear blocks analyses. IR contraction and expansion were studied among these species using IRscope (Amiryousefi et al. 2018a).

We also analyzed synonymous (*K*_s_) and non-synonymous (*K*_a_) substitutions and their ratio (*K*_a_/*K*_s_). *Symplocarpus renifolius*, a species from the early diverging subfamily Orontioideae*,* was used as a reference and 75 protein-coding genes of *P. scandens* and *A. huixtlense* were aligned to the protein-coding genes of *S. renifolius* by MAFFT alignment (Katoh et al. 2005). These alignments were analyzed for the determination of *K*_s_ and *K*_a_ substitutions and K_a_/K_s_ using DnaSP (Rozas et al. 2017) as reported previously (Abdullah et al. 2019a, 2020b; Henriquez et al. 2020a). We further elucidated selective pressure on protein-coding genes using additional codon models such as branch-site unrestricted statistical test for episodic diversification (BUSTED) (Murrell et al. 2015) and fast unconstrained Bayesian AppRoximation (FUBAR) (Murrell et al. 2013) with HyPhy (Pond et al. 2005) using the Datamonkey server (Delport et al. 2010). BUSTED was used with default parameters to investigate diversifying selection on selected genes. FUBAR was used with posterior probability of > 0.9 to identify episodic/diversifying selection on codons sites.

We also determined the extent of transition (*T*_s_) and transversion (*T*_v_) substitutions linked with *K*_s_ and *K*_a_ substitutions. For this purpose, we selected 11 genes from the genome of *P. scandens* that had various *K*_a_/*K*_s_ values and analyzed the extent of transition and transversion types of substitutions with K_s_ and K_a_ substitutions in Geneious R8.1 (Kearse et al. 2012) following Abdullah et al. (2019a).

We analyze the effect of rate heterotachy on the evolution of protein-coding genes, using *S. renifolius* as a reference. We considered genes of LSC, SSC, and IR of *S. renifolius* and determined the rate of evolution of the respective genes in the chloroplast genomes of *P. scandens* and *A. huixtlense*. We also separately determined the rate of evolution of protein-coding genes that were transferred from IRs to LSC or from SSC to IR to elucidate the changes in evolution rate. We concatenated genes of each region and aligned using MAFFT (Katoh et al. 2005). The types of transition and transversion substitutions in *P. scandens* and *A. huixtlense* were also determined from the alignment of genes from LSC, SSC, and IR.

### Phylogenetic Inference

The phylogenetic tree was inferred using 31 species of Araceae, with *Acorus americanus* (Acoraceae) as the outgroup (Table S1). The complete chloroplast genomes, excluding IRa, were aligned by MAFFT (Katoh et al. 2005) and the phylogeny was inferred using the IQ-tree program (Nguyen et al. 2015; Kalyaanamoorthy et al. 2017; Hoang et al. 2018) with default parameters, as reported previously (Abdullah et al. 2019a, 2020b).

## Results

### Assembly and Characterization of Chloroplast Genomes

The sequencing of 100 bp single-end reads generated 3.69 GB data (14.13 million reads) for *A. huixtlense* and 5.8 GB data (22.2 million reads) for *P. scandens*. Whole-genome shotgun reads contained 0.22 million reads in *A. huixtlense* and 0.77 million reads of chloroplast origin in *P. scandens*. These chloroplast reads were used for de novo assembly and provided average coverage depths of 468 × for *P. scandens* and 138 × for *A. huixtlense*.

The sizes of the complete chloroplast genomes were 163,116 bp for *A. huixtlense* and 164,719 bp for *P. scandens*. The sizes of the LSC and SSC regions showed a high level of variation between the two species due to unique IR contraction and expansion in the *P. scandens* chloroplast genome (Table 1). The GC content was highest in IR regions, followed by LSC and SSC regions. A high level of variation exists in the GC content of the chloroplast genome of both species.

**Table 1:** Comparative analyses of chloroplast genomes of *P. scandens* and *A. huixtlense*

Characteristics	*Pothos scandens*	*Anthurium huixtlense*
Size (base pair; bp)	164,719	163,116
LSC length (bp)	102,956	89,260
SSC length (bp)	6779	22,982
IR length (bp)	27,492	25,437
Number of genes	135	130
Protein-coding genes	90	85
tRNA genes	36	37
rRNA genes	8	8
Duplicate genes	22	17
GC content		
Total (%)	35.4	36.2
LSC (%)	34.7	34.5
SSC (%)	28.8	28.5
IR (%)	37.3	42.7
CDS (%)	37.4	38
rRNA (%)	54.9	55
tRNA (%)	53.3	53.2

**Fig. 1:**
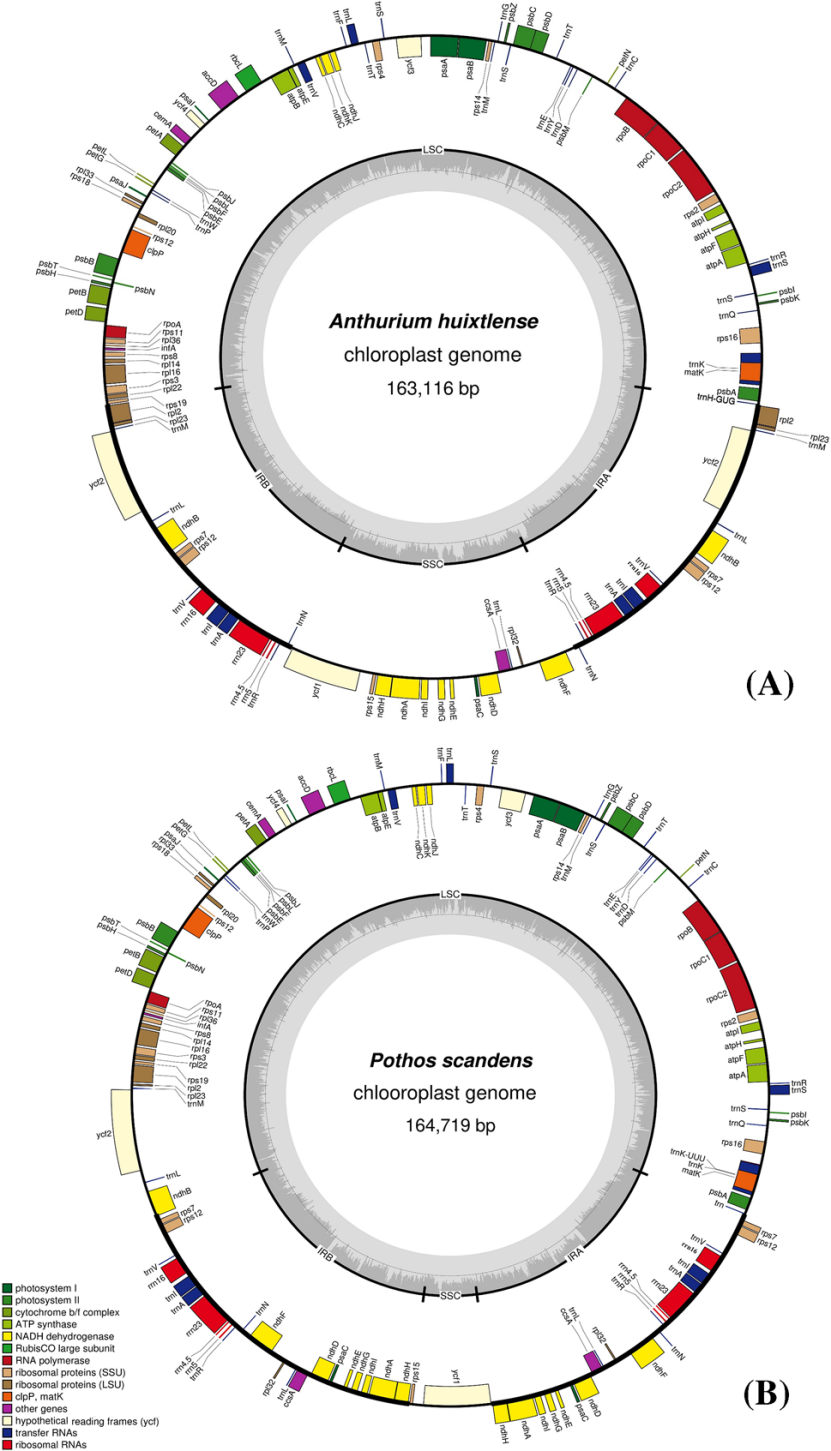
Circular diagram of chloroplast genomes of *A. huixtlense* and *P. scandens*. LSC, SSC, and IR represent large single-copy, small single-copy, and inverted repeat regions, respectively. The genes located inside are transcribed counterclockwise, whereas the genes located outside are transcribed clockwise

We found 113 unique functional genes in both species, including 79 protein-coding genes, 30 tRNA genes, and 4 rRNA genes (Fig. 1a, b). The *inf*A gene was observed as a pseudogene in both species, whereas the *rpl*23 gene was observed as pseudogene in *P. scandens* due to the generation of an internal stop codon. The total number of genes varied between the two species due to IR contraction and expansion. We found 130 genes in *A. huixtlense*, including 37 tRNA genes, 85 protein-coding genes, and 8 rRNA genes. We also observed 17 genes that were duplicated in the IR regions in *A. huixtlense*, including 7 tRNA genes (2 genes also contain introns), 4 rRNA genes, and 6 protein-coding genes (3 genes also contain introns) (Fig. 1a). In *P. scandens*, we found 135 genes due to expansion of the IR region, including 36 tRNA genes, 90 protein-coding genes, and 8 rRNA genes (Fig. 1b). We found 22 genes that were duplicated in the IR regions in *P. scandens*, including 6 tRNA genes (2 genes also contain introns), 4 rRNA genes, and 12 protein-coding genes (2 genes also contain introns) (Fig. 1b).

### Amino Acid Frequency and Codon Usage

**Fig. 2:**
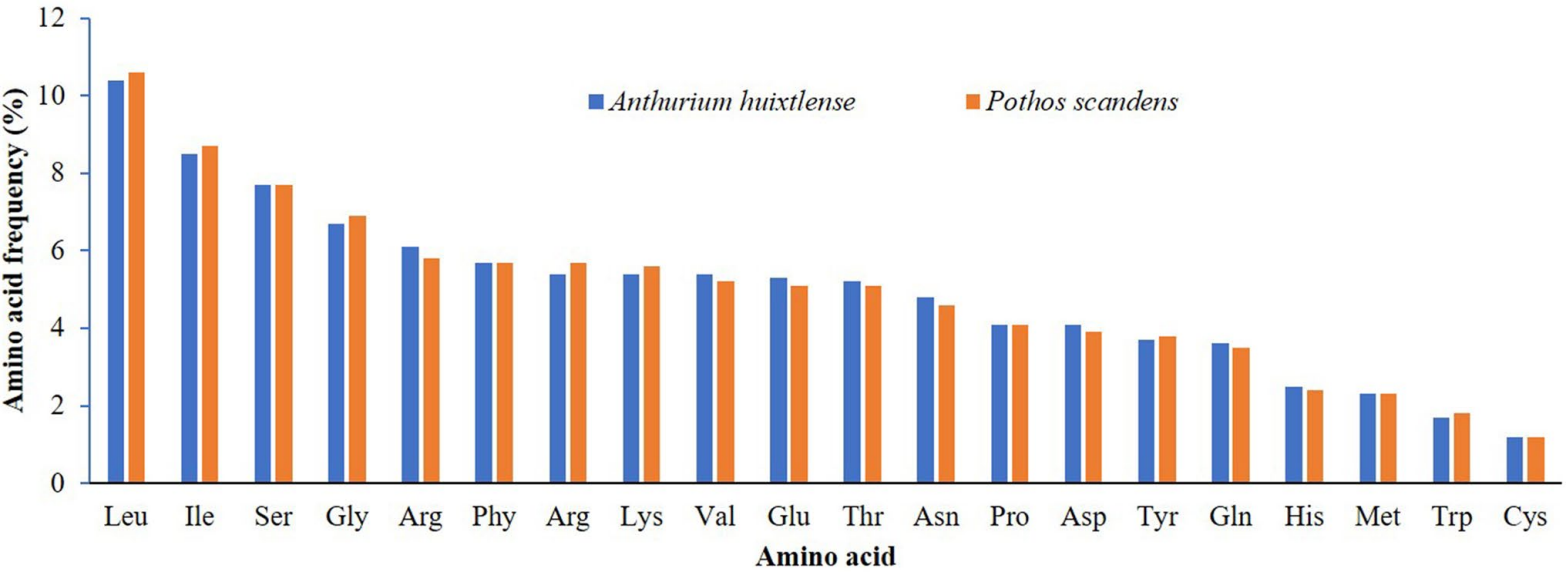
Amino acid frequency in *A. huixtlense* and *P. scandens*. The* x*-axis shows amino acids, whereas the* Y*-axis shows percentage of amino acid frequency

The highest frequency observed was for leucine followed by iso-leucine, whereas the lowest frequency observed was for cysteine (Fig. 2). Relative synonymous codon usage (RSCU) analyses revealed high encoding frequency for codons containing A or T at the 3′ end and having an RSCU value of ≥ 1, whereas low encoding frequency was observed for codons having C or G at the 3′ and having RSCU < 1 (Table S2).

### Repeats Analyses

**Fig. 3:**
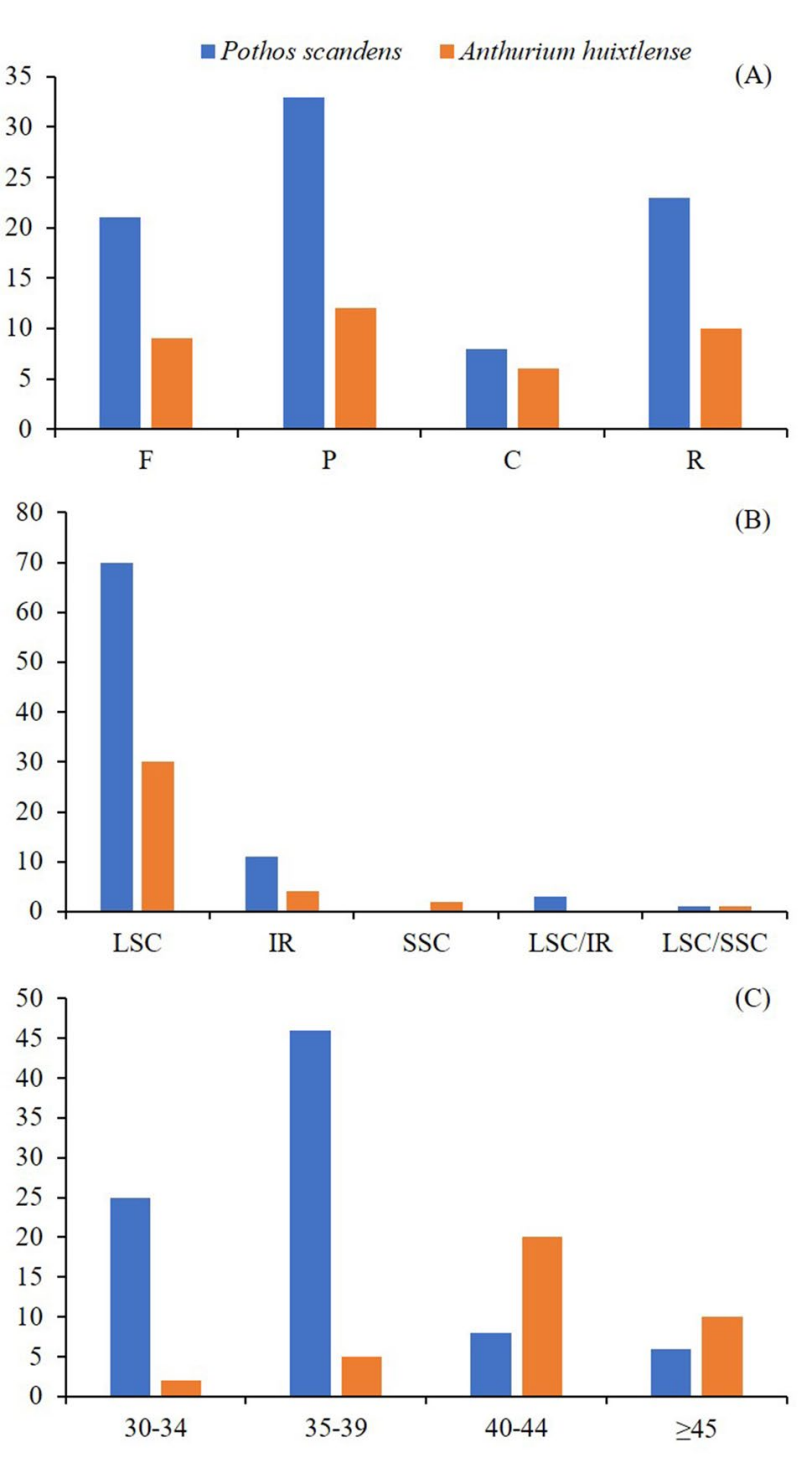
Analyses of repeats in *A. huixtlense* and *P. scandens*. **a** Types of repeats. **b** Distribution of repeats in the chloroplast genomes. **c** The size of repeats in the genome. *F* Forward, *C* complementary, *R* reverse, *P* palindromic, *LSC* large single copy, *SSC* small single copy, *IR* inverted repeat, LSC/SSC and LSC/IR represent shared repeats between regions

REPuter detected four types of oligonucleotide repeats in the chloroplast genomes of *A. huixtlense* and *P. scandens*. The number of repeats and types varied in both species to a high degree. We observed 37 repeats in *A. huixtlense* and 85 repeats in *P. scandens*. We observed 9 forward, 12 palindromic, 6 complementary, and 10 reverse repeats in *A. huixtlense*. In *P. scandens* we observed 21 forward, 33 palindromic, 8 complementary, and 23 reverse repeats (Fig. 3a). Most of the repeats were found in LSC regions instead of SSC and IR regions (Fig. 3b). Most of the repeats ranged in size from 40 to 44 bp in *A. huixtlense*. In *P. scandens*, most of the repeats varied in size from 35 to 39 bp (Fig. 3c). Details are provided in Table S3.

### Evolutionary Rate of Protein-Coding Genes

The evolution rate of protein-coding genes revealed strong purifying selection on these genes and that none of the genes are under positive selection pressure. Except for a few genes that showed neutral selection, all other genes showed purifying selection (Table S4) (average *K*_s_ = 0.16, *K*_a_ = 0.026, and *K*_a_/*K*_s_ = 0.18). As expected, the highest purifying selection pressure was observed for genes that are involved in photosynthesis.

The results of codon models were similar to the *K*_a_/*K*_s_ method, and none of the genes was found to be under positive selection in the subfamily Pothoideae using BUSTED. The mixture model implemented in BUSTED needs a relatively high fraction of sites (5–10%) to be under positive selection for accurate detection. At a next step, we implemented FUBAR to detect rare sites that might be under positive selection. These tests showed a few codons under positive selection in genes *rpl*22 (2 codons), *ycf*1 (8 codons), and *ycf*2 (5 codons) (Table S5).

In the protein-coding genes of *P. scandens*, we found 4061 substitutions in comparison with *Symplocarpus renifolius* reference genome. Of these, 2814 contained transition (T_s_) substitutions and 1247 contained transversion (T_v_) substitutions; the *T*_s_/*T*_v_ ratio was 2.26. In *A. huixtlense*, we recorded 3960 substitutions, of which 2690 were *T*_s_ and 1270 were *T*_v_; the *T*_s_/*T*_v_ ratio was 2.12 (Table 2). Examination of 11 protein-coding genes revealed a *T*_s_/*T*_v_ of 2.79 for synonymous substitutions and a *T*_s_/*T*_v_ of 1.43 for non-synonymous substitutions. Hence, a higher number of *T*_v_ leads to non-synonymous substitutions as compared to *T*_s_ and vice versa.

**Table 2:** Transition and transversion substitutions in protein-coding genes of *P. scandens and A. huixtlense*

SNP type	*P. scandens*	*A. huixtlense*
C/T	1416	1331
A/G	1398	1359
A/T	255	250
C/G	157	144
G/T	344	358
A/C	491	518

### Gene Arrangement and Inverted Repeats Contraction and Expansion

**Fig. 4:**
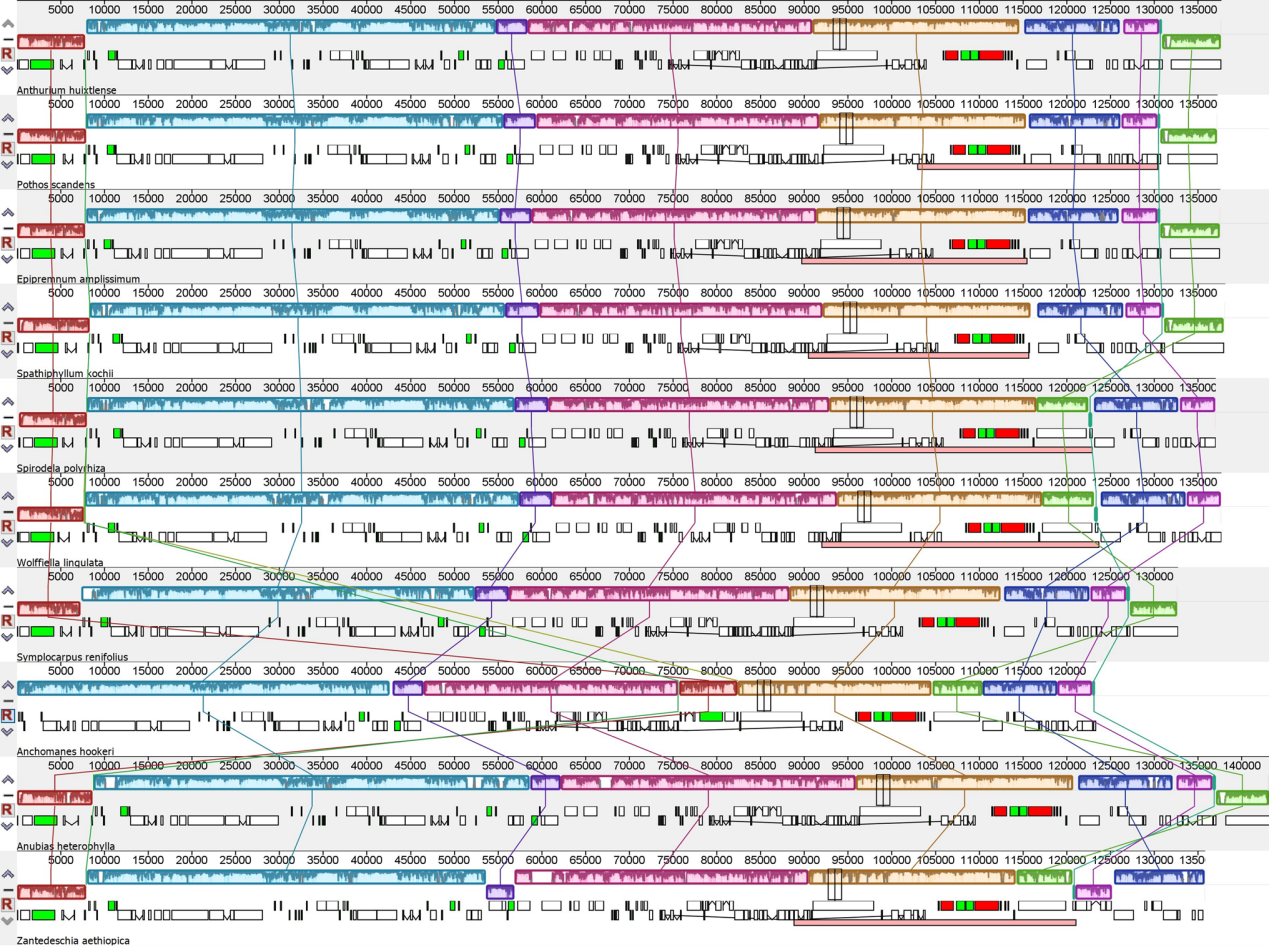
Analyses of gene arrangements among the species of Araceae based on Mauve alignment. White boxes indicate protein-coding genes, red indicate rRNA, black indicate tRNA, green indicate intron-containing tRNA, and the line between two white boxes indicates intron-containing protein-coding genes

The genomes of Pothoideae show unique gene and structural rearrangements. The *P. scandens* chloroplast genome showed unique IR contraction and expansion, which led to a variable number of genes and also a change in gene arrangement. At the JLB junction (LSC/IRb), the contraction of IR resulted in expansion of the LSC region, whereas at the JSB (IRb/SSC) junction, the expansion of IR resulted in contraction of the SSC region. Hence, genes including *rpl*2, *rpl*23, *trn*M, *ycf*2, *trn*L, *ndh*B that are usually duplicated in the IRs became single copy after their transfer to LSC. In contrast, genes such as *ndh*H, *ndh*A, *ndh*I, *ndh*G, *ndh*E, *psa*C, *ndh*D, *ccs*A, *trn*L, *rpl*32, and *ndh*F that usually exist as single copy in SSC were duplicated after their transfer to IR regions (Fig. 1b). The arrangement of genes in LSC in both *A. huixtlense* and *P. scandens* did not change due to contraction of IR regions and gene arrangement was found to be similar to other species (*Spathiphyllum kochii*, *E. amplissimum*, *S. renifolius*, and *A. heterophylla*), as shown in Colinear block of Mauve alignment (Fig. 4). However, the genes of the SSC region showed variation in gene arrangement (Fig. 4). In the genome of *A. huixtlense*, the SSC was inverted when compared to other species of Aroideae. However, this could not be considered an important evolutionary event as chloroplast genomes exist in two equimolar states and can be differentiated by orientation of the SSC region (Walker et al. 2015).

**Fig. 5:**
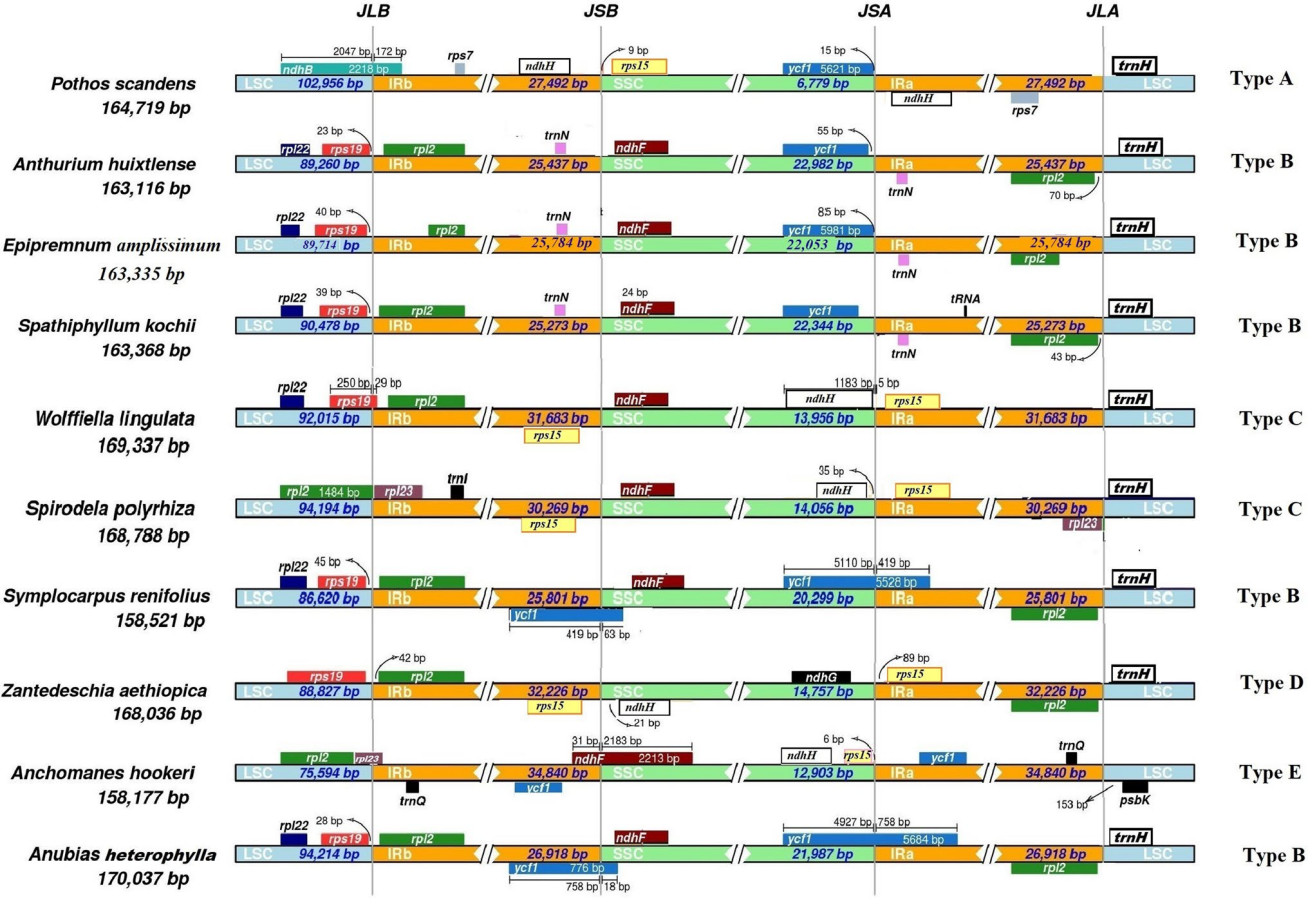
Comparative analysis of junction sites in Araceae chloroplast genomes. Abbreviations denote junction site of the plastid genome JLA (IRa/LSC), JLB (IRb/LSC), JSA (SSC/IRa) and JSB (IRb/SSC). Genes are represented by colored boxes while arrows are showing the coordinate positions of each gene near the junctions. Genes displayed on the top appear on the negative strand, while genes present bellow and found on the positive strand of the genome

The contraction and expansion of IR regions at the junctions JLB (LSC/IRb), JSB (IRb/SSC), JSA (SSC/IRa), and JLA (IRa/LSC) were analyzed among the species of Araceae. We observed five types of variation in the junctions (Fig. 5). Type A included *P. scandens*, type B included *A. huixtlense*, *E. amplissimum*, *S. kochii*, *S. renifolius*, and *A. heterophylla*, type C included *Wolffiella lingulata* and *Spirodela polyrhiza*, type D included *Z. aethiopica*, and Type E included *A. hookeri*. These results show that the *P. scandens* chloroplast genome displays a novel type of IR contraction and expansion.

### Effects of Rate Heterotachy

Contraction and expansion in IRs affected the rate of evolution in protein-coding genes. The genes that were transferred from the SSC region to IR regions showed a decrease in the rate of evolution, whereas genes that were transferred from IR regions to the LSC region showed an increase in the rate of evolution. In *P. scandens*, we found 2454 (5.67%) substitutions in the genes located in LSC, 269 substitutions (2.64%) in the genes present in IRs, and 1338 (9.27%) substitutions in the genes found in SSC. In *A. huixtlense*, we found 2428 (5.62%) substitutions in genes of LSC, 205 (2.0%) substitutions in genes of IRs, and 1327 (9.16%) in genes of SSC. We found a higher rate of evolution in *P. scandens* genes than in *A. huixtlense* and observed a difference of 0.043% in genes of LSC, 0.64% in genes of IRs, and 0.11% in genes of SSC. We observed the highest difference in evolution rate between *P. scandens* and *A. huixtlense* in IRs. This might be due to transfer of most of the IR genes of *P. scandens* to LSC region. To further verify the effect of rate heterotachy, we separately compared the rate of evolution of those genes that transferred from SSC to IRs in *P. scandens*. Genes of *P. scandens* that were transferred from SSC to IRs showed 0.43% less evolution than genes of *A. huixtlense*, whereas average rate of evolution of the genes of all regions were found higher in *P. scandens* than in *A. huixtlense*. This confirmed that the transfer of the genes from single-copy regions to IRs is responsible for decreased evolution rates.

### Phylogenetic Inference of the Family Araceae

**Fig. 6:**
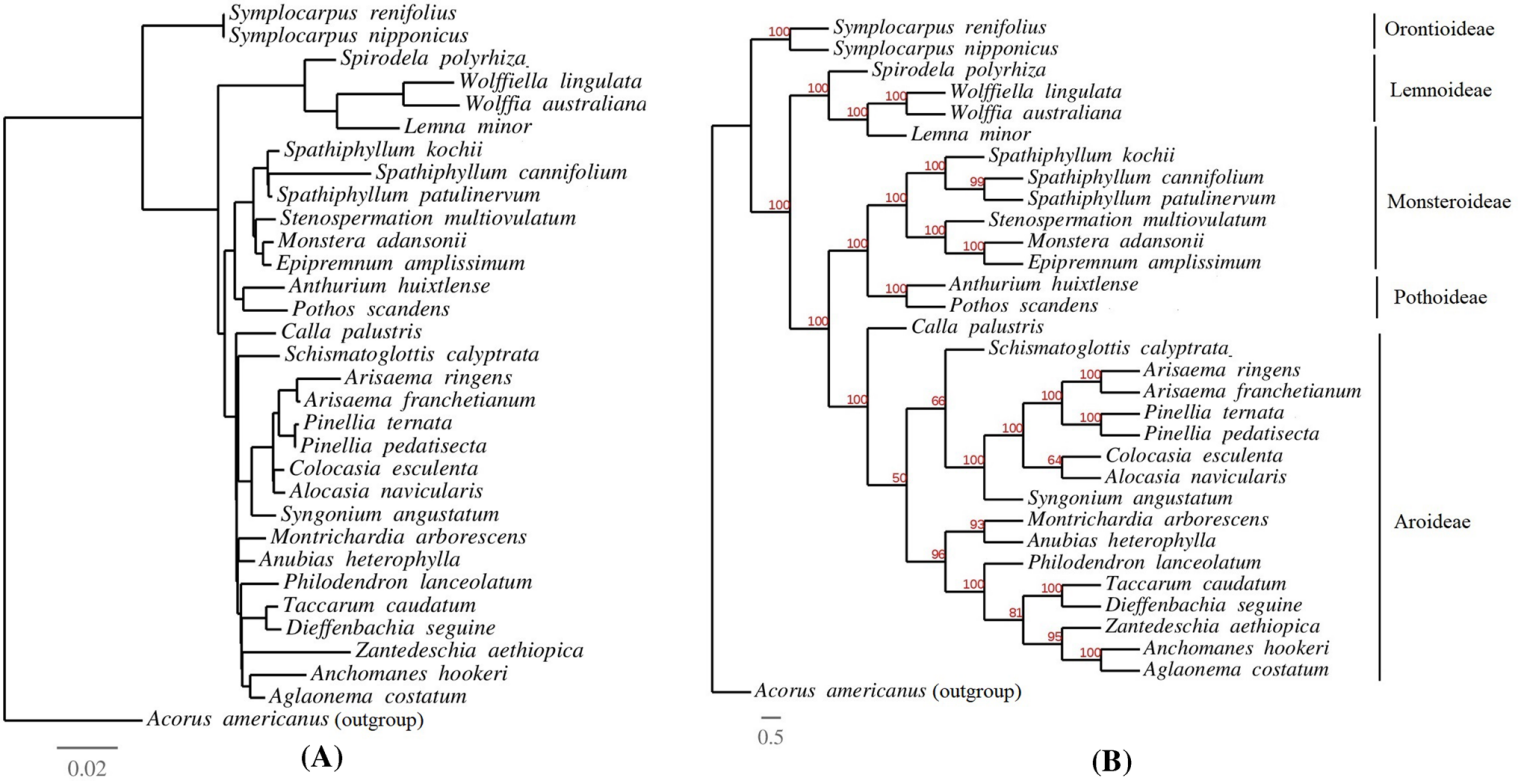
Maximum likelihood phylogenetic tree of the Araceae family reconstructed from plastid genome data. **a** Phylogenetic tree and **b** cladogram

The phylogenetic tree was reconstructed with the best fitting Model GTR + F + I + G4. The nucleotide alignment contained a total of 94,654 sites in which 65,927 were invariable, 14,393 were parsimony informative, and 9262 sites showed a distinct pattern. The phylogenetic tree supported the monophyly of the five subfamilies that were included in the study with 100% bootstrap support (Fig. 6). However, at subfamily level, the low bootstrap support was also observed for some nodes, specifically among the species of subfamily Aroideae (Fig. 6). The subfamily Pothoideae showed sister relationship with the subfamily Monsteroideae. The subfamily Orontioideae was the basal group, whereas Aroideae was the crown group.

## Discussion

In the current study, we assembled the chloroplast genomes of two species from subfamily Pothoideae of Araceae. The chloroplast genomes of both *P. scandens* and *A. huixtlense* were found to be unique among Araceae species and showed a unique type of IR contraction and expansion that affected the rate of evolution in *P. scandens*.

In the current study, the chloroplast genome of *P. scandens* showed uneven IR contraction and expansion, which led to a variable number of genes. IR contraction and expansion is very common in chloroplast genomes and leads to variation in the number of genes in various plant lineages, including Araceae (Ahmed et al. 2012; Menezes et al. 2018; Cho et al. 2018; Lee et al. 2018; Abdullah et al. 2020b; Henriquez et al. 2020b). IR contraction and expansion also results in new combinations of genes in the IR regions, which in turn leads to rearrangement of genes in the SSC region, as previously reported in Araceae (Wang and Messing 2011; Ahmed et al. 2012; Henriquez et al. 2020b). However, in *P. scandens*, we observed the formation of a new combination of genes in IRs but not an accompanying rearrangement of the genes. A similar effect of IR contraction and expansion was also reported in other plant lineages without any effect on the arrangement of genes (Wang et al. 2016; Cho et al. 2018; Lee et al. 2018). In *P. scandens*, the SSC region showed significant shortening and contained only two genes. Similar shortening of the SSC region was also reported in other angiosperms and even smaller SSC regions have been reported (Cho et al. 2018; Lee et al. 2018). Previously, four types of gene arrangements were observed in Araceae. Two types of gene arrangements were observed at IR junctions in one comparison of Araceae species (Choi et al. 2017) and two other types of gene arrangements at the junctions were reported in the chloroplast genomes of two species of subfamily Aroideae, including *A. hookeri* and *Z. aethiopica* (Henriquez et al. 2020b). In the current study, we reported a fifth type of gene arrangement at the junctions in the chloroplast genome of *P. scandens*. Further genomic resources from the genus *Pothos* and subfamily Pothoideae might be helpful to gain insight into chloroplast genome structure and to discern whether this uneven IR contraction and expansion occurs only in *P. scandens* or in the genus *Pothos* as a whole. The reason of IR contraction and expansion might be due to double strand break model and illegitimate recombination, as previously suggested in Mimosoideae (Wang et al. 2017).

The expansion of IR regions in the *P. scandens* genome reported here decreased the evolutionary rate of protein-coding genes that shifted from SSC to IR, whereas an increase in the evolutionary rate was observed in the genes that transferred from IR to LSC. Similar results were reported in the chloroplast genomes of other species and a higher rate was observed in the regions that exist in the single-copy region instead of IR region (Zhu et al. 2016). In contrast, the effect on evolutionary rate in *Pelargonium* was not observed due to IR contraction and expansion (Weng et al. 2017). This phenomenon might have been due to incipient transfer of genes in *Pelargonium*. The low rate of evolution in genes that exist in IR regions might be due to a repairing mechanism (Zhu et al. 2016).

The variations in chloroplast genomes are important for the studies of population genetics and phylogenetics and provide insight into evolutionary relationship of various taxa (Ahmed 2014; Henriquez et al. 2014). The polymorphisms of chloroplast genomes are also helpful to gain insight into the origin, geographical distribution, domestication and adaptation of plants to various climatic conditions (Daniell et al. 2016). Moreover, the polymorphisms of chloroplast genomes are useful in the identification of commercial cultivars (Suzuki et al. 2017), and identification of closely related and genetically compatible species for breeding (Daniell et al. 2016). Previously, *ycf*1 region was used for phylogenetic inference in the subfamily Monsteroideae (Zuluaga et al. 2019). Our study demonstrates that the rate of evolution for the genes at the junctions of single-copy and IR regions are affected by rate heterotachy. The utilization of such regions is amenable to misleading results in drawing phylogenetic inferences (Lockhart et al. 2006; Zhong et al. 2011). Hence, such genes should be avoided while studying phylogenetic relationships and determining times of divergence when using few loci. Genes which are important in domestication and adaption remain functional during stress conditions, despite occurrence of long inversion and high level of IR contraction and expansion (Daniell et al. 2016). In our study, no gene loss was evident despite significant contraction of SSC and expansion of IRs in *P. scandens* which provide insight into the important role of these genes in adaptation.

Chloroplast genomes are mostly conserved in terms of gene content and organization, and GC content of LSC, SSC and IR regions (Wang and Messing 2011; Ahmed et al. 2012; Iram et al. 2019; Abdullah et al. 2020b; Henriquez et al. 2020a,b; Shahzadi et al. 2020). We observed high GC content in the IR regions when compared with the LSC and SSC regions, consistent with previous reports. However, the IR regions of the *P. scandens* genome showed a decrease in GC content up to 5% when compared with *A. huixtlense* genome. This was due to expansion of the IR regions and subsequent inclusion of most of the genes of SSC region (which has low GC content). Average GC content of the regions of SSC that were transferred to IRs was 29.3%, whereas the average GC content of IRs genes was as high as 43%.

In our study, leucine and iso-leucine remained the most frequent, while cysteine remained the least frequent amino acid. Higher RSCU values (≥ 1) were found for codons with A or T at the 3′ position and showed high encoding efficacy. Similar results for amino acid frequency and codon usage have also been reported in the chloroplast genomes of other angiosperms, which might be due to the high overall AT content in the chloroplast genome (Amiryousefi et al. 2018b; Menezes et al. 2018; Abdullah et al. 2019b; Mehmood et al. 2020). The analyses of oligonucleotide repeats showed the existence of four types of repeats, but the repeats varied in size and types between the two species. The variation in the types and size of repeats were also previously reported in the chloroplast genomes of angiosperms and in other species of Araceae (Abdullah et al. 2020b; Henriquez et al. 2020b; Mehmood et al. 2020). These repeats might be useful as a proxy to identify mutational hotspots (Ahmed et al. 2012; Abdullah et al. 2020c) and design molecular markers for phylogenetic and population genetics studies (Ahmed et al. 2013).

Our finding of increased transition substitutions compared to transversion substitutions is consistent with other findings (Wakeley 1996; Cao et al. 2018). Contradictory findings of more transversion than transition findings have also been documented previously (Cai et al. 2015; Abdullah et al. 2019a; Shahzadi et al. 2020). This bias of higher transversions might be due to the composition of genomes and the genetic characteristics of codons (Morton et al. 1997). We observed higher transition substitutions linked to synonymous substitutions and vice versa, as reported in the chloroplast genomes of *Firmiana*, a genus of family Malvaceae (Abdullah et al. 2019a).

The higher rate of synonymous substitutions than non-synonymous substitutions is indicative of strong purifying selection pressure acting on these genes during the course of evolution (Matsuoka et al. 2002). The observation of purifying selection in our study are consistent with previous studies on angiosperm chloroplast genomes, including other aroid (Menezes et al. 2018; Abdullah et al. 2019a, 2020a, b; Henriquez et al. 2020a; Shahzadi et al. 2020). In current study, some codons sites in three protein-coding genes (*rpl*22, *ycf*1, and *ycf*2) were found under positive selection pressure in the subfamily Pothoideae based on the results of FUBAR. The *rpl*22 is encoding the large subunit of ribosomes (Mache 1990) while *ycf*1 and *ycf*2 are the largest protein-coding genes in the chloroplast genome that are part of the chloroplast inner envelope membrane protein translocon (Kikuchi et al. 2013). These genes were also found under positive selection in species of Araceae and other angiosperms (Fan et al. 2018; Liu et al. 2018; Zhong et al. 2019; Henriquez et al. 2020a; Abdullah et al. 2020a). The presence of positively selected codons revealed the role of these genes in adaptation of the species in their ecological niches. Previously, only seven genes in subfamily Monsteroideae were found under positive selection (Henriquez et al. 2020a), whereas in comparison of chloroplast genomes of four species from the three subfamilies of Araceae three genes were reported under positive selection (Abdullah et al.  2020a). In another study of Araceae, most genes (62/71) were reported under positive selection (Kim et al. 2019) based on the evaluation of *K*_a_/*K*_s_ values in DnaSP. We also followed the *K*_a_/*K*_s_ methodological approach of Kim et al. (2019). However, Kim et al. (2019) included 13 other species from four subfamilies of Araceae (Orontioideae, Lemnoideae, Monsteroideae, and Aroideae) and a misidentified taxon, *Alocasia macrorrhizos* (L.) G.Don (KR296655) in their comparison. The inclusion of these additional taxa might be the reason for the observation of unusually high number of genes undergoing positive selection, reported by Kim et al. (2019); as positive selection was also reported on 1/3 protein-coding genes of grasses (Piot et al. 2018).

The phylogenetic inference among the species of five subfamilies (Orontioideae, Lemnoideae, Pothoideae, Monsteroideae, and Aroideae) of Araceae are in agreement with previous findings (Cabrera et al. 2008; Cusimano et al. 2011; Nauheimer et al. 2012; Henriquez et al. 2014). Kim et al. (2019) showed an unusual sister relationship between *Epipremnum* (Monsteroideae) and *Dieffenbachia* (Aroideae). In our study, *Epipremnum* was confirmed to share a sister relationship with the genus *Monstera* in subfamily Monsteroideae instead of Aroideae. Hence, phylogenetic inference based on complete chloroplast genome confirmed the placement of genus *Epipremnum* in Monsteroideae as reported previously (Cusimano et al. 2011; Henriquez et al. 2014; Chartier et al. 2014; Zuluaga et al. 2019). Kim et al. (2019) included *Epipremnum aureum* (MK286107) in their comparison, hence, this shows that *E. aureum* is either mislabeled or is a case of chloroplast genome capture from another species as hybridization and polyploidy is common in Araceae (Ahmed 2014).

In conclusion, our study provides insight into the evolution of chloroplast genomes of Pothoideae (Araceae). Our study shows unique IR contraction and expansion affecting the number of genes and rate of evolution in *P. scandens*. We observed a two-fold higher transition substitution rate than transversion substitutions and found higher transversion substitutions linked with non-synonymous substitutions when compared with transition substitutions.

## Electronic supplementary material

Below is the link to the electronic supplementary material.

Supplementary file1 (DOCX 15 kb)

Supplementary file2 (XLSX 14 kb)

Supplementary file3 (XLSX 15 kb)

Supplementary file4 (XLSX 17 kb)

Supplementary file5 (XLSX 10 kb)
