# Supplementary material for: Complete Chloroplast Genomes of Anthurium huixtlense and Pothos scandens (Pothoideae, Araceae): Unique Inverted Repeat Expansion and Contraction Affect Rate of Evolution
Source: J Mol Evol. 2020 Jul 9;88(7):562–74. doi: 10.1007/s00239-020-09958-w (PMC7445159; doi:10.1007/s00239-020-09958-w)
Supplement: Supplementary file 1 — Supplementary file1 (DOCX 15 kb) [file 239_2020_9958_MOESM1_ESM.docx]

Table S1. GenBank accession number of the species used in phylogenetic inference

| S. No | Species | Accession |
| --- | --- | --- |
| 1 | *Acorus americanus (outgroup)* | EU273602 |
| 2 | *Colocasia esculenta* | JN105689 |
| 3 | *Lemna minor* | DQ400350 |
| 4 | *Spirodela polyrhiza* | JN160603 |
| 5 | *Wolffiella lingulata* | JN160604 |
| 6 | *Wolffia australiana* | JN160605 |
| 7 | *Spathiphyllum kochii* | KR270822 |
| 8 | *Pinellia ternata* | KR270823 |
| 9 | *Symplocarpus renifolius* | KY039276 |
| 10 | *Arisaema ringens* | MK111107 |
| 11 | *Dieffenbachia seguine* | NC_027272 |
| 12 | *Anubias heterophylla* | MN046884 |
| 13 | *Arisaema franchetianum* | MN046885 |
| 14 | *Schismatoglottis calyptrata* | MN046892 |
| 15 | *Pinellia pedatisecta* | MN046890 |
| 16 | *Zantedeschia aethiopica* | MN551189 |
| 17 | *Philodendron lanceolatum* | MN551187 |
| 18 | *Taccarum caudatum* | MN046895 |
| 19 | *Montrichardia arborescens* | MN046889 |
| 20 | *Aglaonema costatum* | MN046881 |
| 21 | *Alocasia navicularis* | MN046882 |
| 22 | *Syngonium angustatum* | MN046894 |
| 23 | *Anchomanes hookeri* | MN551188 |
| 24 | *Calla palustris* | MN046887 |
| 25 | *Symplocarpus nipponicus* | MK341566 |
| 26 | *Spathiphyllum patulinervum* | MN046890 |
| 27 | *Spathiphyllum cannifolium* | MK372232 |
| 28 | *Stenospermation multiovulatum* | MN046893 |
| 29 | *Epipremnum amplissimum* | MN046885 |
| 30 | *Monstera adansonii* | MN046888 |
